# Deciphering Physiological Functions of AHL Quorum Quenching Acylases

**DOI:** 10.3389/fmicb.2017.01123

**Published:** 2017-06-19

**Authors:** Putri D. Utari, Jan Vogel, Wim J. Quax

**Affiliations:** Chemical and Pharmaceutical Biology Department, University of GroningenGroningen, Netherlands

**Keywords:** *N*-acylhomoserine lactones, acylases, amidases, quorum sensing, quorum quenching, Ntn-hydrolases

## Abstract

*N*-Acylhomoserine lactone (AHL)-acylase (also known as amidase or amidohydrolase) is a class of enzyme that belongs to the Ntn-hydrolase superfamily. As the name implies, AHL-acylases are capable of hydrolysing AHLs, the most studied signaling molecules for quorum sensing in Gram-negative bacteria. Enzymatic degradation of AHLs can be beneficial in attenuating bacterial virulence, which can be exploited as a novel approach to fight infection of human pathogens, phytopathogens or aquaculture-related contaminations. Numerous acylases from both prokaryotic and eukaryotic sources have been characterized and tested for the interference of quorum sensing-regulated functions. The existence of AHL-acylases in a multitude of organisms from various ecological niches, raises the question of what the physiological roles of AHL-acylases actually are. In this review, we attempt to bring together recent studies to extend our understanding of the biological functions of these enzymes in nature.

## Introduction

**FIGURE 1:**
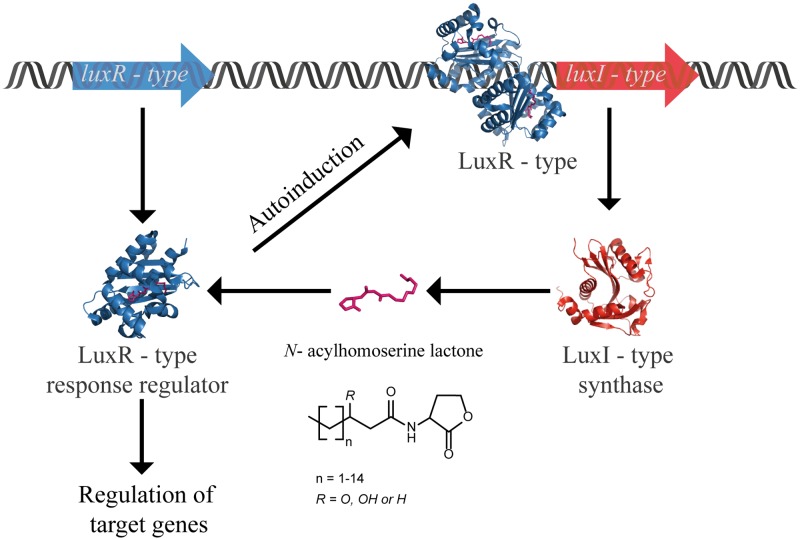
Typical *N*-acylhomoserine lactone-dependent QS core circuit in Gram-negative bacteria. The LuxI-type synthase homologs produce AHLs, the signaling molecules consist of a homoserine lactone ring linked via an amide bond to an acyl chain with a variety of chain lengths and substitutions. AHLs are recognized by LuxR-type homologs, the transcriptional regulators that are composed of two domains: an N-terminal domain that binds to AHL, and a C-terminal domain that recognizes a specific DNA sequence. Following binding to the AHL, the regulator proteins will undergo dimerization prior to transcriptional activation. This in turn affects the expression level of several genes regulated by QS including the AHL synthase, allowing an up regulation or a down regulation depending on the bacterial model. The protein structures in this figure are represented by LasI (PDB file: 1RO5) and LasR (PDB file: 3IX3) from *P. aeruginosa*.

Quorum sensing (QS) is a cell-to-cell communication that allows bacteria to synchronize collective behavior in a population density-dependent manner. This unique social interaction is often related to the regulation of highly complex structures and goods that are metabolically too expensive, if not impossible, to be produced by an individual cell (Bassler and Losick, 2006). In Gram-negative bacteria, QS depending on *N*-acylhomoserine lactones (AHLs) autoinducers is the most well-known communication system. The typical core system consists of the production and secretion of autoinducers, followed by a signal recognition by transcriptional regulators that subsequently regulate expression of QS-related genes, including those of autoinducer synthases (**Figure 1**). Variations of this system exist in diverse Gram-negative bacterial models (Papenfort and Bassler, 2016). The autoinducer synthases (LuxI homologs) and the transcriptional regulators (LuxR homologs) often can synthesize and perceive multiple AHLs, respectively (Williams, 2007).

Quorum sensing not only matters for communication within one species, but is also important for making connections with a bigger social network that involves other bacterial species (*interspecies*) and organisms from different kingdoms (*interkingdom*), because they reside alongside each other or are in symbiosis in nature. There is growing evidence that cross-communication involving QS is existing in highly complex communities composed of multiple organisms. For instance, the lungs of cystic fibrosis patients are often colonialized by multiple pathogens. The predominant pathogen *Pseudomonas aeruginosa* produces 3OC12-HSL that not only regulates QS in another pathogen (Eberl and Tümmler, 2004), but also shows immunomodulatory activities toward the host. In a dose-dependent manner, 3OC12-HSL directly elicits the production of a neutrophil-attracting chemokine, interleukin 8 (IL-8), in human lung fibroblasts and epithelial cell lines (DiMango et al., 1995; Smith et al., 2001). However, longer exposure to higher concentrations of 3OC12-HSL can promote an adverse effect by accelerating apoptosis in neutrophils and macrophages (Telford et al., 1998; Tateda et al., 2003; Li et al., 2004). On the other hand, interkingdom mutualism is also common, one well-known example is an interaction between legumes family with its symbionts, the nitrogen-fixating bacteria called rhizobia. AHL-dependent QS systems in several members of *Rhizobiaceae* have been found to influence the symbiotic interactions (Sanchez-Contreras et al., 2007; Hartmann et al., 2014). An AHL from *Sinorhizobium meliloti*, 3O14-HSL, benefits the bacteria by inducing an increase in the nodulation of the model legume *Medicago truncatula* (Veliz-Vallejos et al., 2014). Hence, mutation of the *lux* homolog locus of *S. meliloti* leads to a decrease and delay of nitrogen-fixation nodules production, demonstrating a role of QS in establishing the symbiosis (Marketon et al., 2002).

**FIGURE 2:**
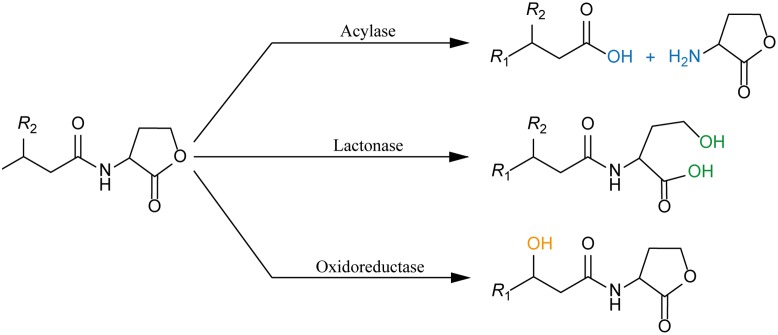
AHL-deactivating enzymes: Three classes of enzymes are categorized as AHLs QQ enzymes, namely: (i) acylase (also known as amidase or aminohydrolase) that hydrolases the amide bond between the acyl chain and the homoserine lactone ring (blue); (ii) lactonase that opens the homoserine lactone ring (green); and (iii) oxidoreductase that modifies the AHLs by oxidizing or reducing the acyl chain without degrading the AHLs (orange).

Numerous molecules in nature can disarm QS by blocking different steps of the signaling pathway, in an interference called quorum quenching (QQ). This perturbation can be provoked by (i) inhibition of LuxI ending the synthesis of AHL, (ii) inhibition of LuxR inactivating the transcription factor, and (iii) degradation of the signal molecule. The latter can be achieved by enzymatic inactivation of AHLs. Three classes of enzymes are categorized as AHLs QQ enzymes, namely: (i) acylase (also known as amidase or aminohydrolase) that hydrolases the amide bond between the acyl chain and the homoserine lactone ring; (ii) lactonase that opens the homoserine lactone ring; and (iii) oxidoreductase that modifies the AHLs by oxidizing or reducing the acyl chain at the third or distal carbon without degrading the AHLs (**Figure 2**). With respect to the demand for new antibiotics due to the alarming emergence of drug-resistance strains, it is understandable why the majority of QQ researches are heavily devoted to exploit QQ agents to fight infections (Chen et al., 2013; LaSarre and Federle, 2013; Fetzner, 2014; Grandclément et al., 2015). In many pathogens, QS plays a crucial role in the regulation of virulence factors production that often determine the success of establishing an infection in the host. Hence, blocking QS renders the bacteria less virulent, and is seen as a prospective strategy to develop novel therapies that will provoke less pressure for resistance. On the other side, taking into account the widespread occurrence of QQ enzymes in numerous organisms, the fundamental knowledge of the physiological function of these molecules is intriguing and has to be addressed.

This review aims to bring together the recent publications relating to the biological functions of the QQ enzymes, focusing to the AHL-acylases. The possible roles of AHL-acylases with and without relation to QS modulation are being discussed, including a comparison with the known functions of other classes of QQ enzymes.

## Quorum Quenching Acylases

### N-terminal Nucleophile Hydrolase Superfamily

Most of the known bacterial AHL-acylases are members of the N-terminal nucleophile (Ntn)-hydrolase, a superfamily of enzymes that was coined by Brannigan et al. (1995). This group consists of diverse enzymes that recognize different kinds of substrates, but share common properties such as an autocatalytic activation, the presence of an N-terminal catalytic nucleophile (threonine, serine, or cysteine) and cleavage of amide bonds (Oinonen and Rouvinen, 2000). Members of this superfamily amongst others are glutamine amidotransferase (Isupov et al., 1996), aspartylglucosaminidase (AGA) (Guan et al., 1998), the 20S proteasome (Groll et al., 1997), and penicillin acylase (Schumacher et al., 1986). The latter is one of the most prominent and best characterized representatives of the Ntn-Hydrolases. For decades, this enzyme is in focus of the scientific community as well as biotech companies (Arroyo et al., 2003). It catalyzes the deacylation of β-lactam antibiotics, producing 6-aminopenicillanic acid, a base intermediate for many semi-synthetic antibiotics (Hewitt et al., 2000). Penicillin acylases are classified based on their substrate specificity, as penicillin G acylase (PGA) and penicillin V acylase (PVA), with benzylpenicillin and phenoxymethylpenicillin as their preferential substrate, respectively (Sio and Quax, 2004).

The sequence similarity within the Ntn-hydrolase family is very low and the enzymes within this family exhibit quaternary structures ranging from heterodimers (PGAs), homotetramers (PVAs) to the complex four homoheptameric rings forming the 20S proteasome (Unno et al., 2002; Mukherji et al., 2014; Sunder et al., 2016). However, a conservation of their tertiary fold structure is strongly pointing toward their evolutionary relationship (Hewitt et al., 2000). The active sites and core structures of the enzymes are located in typically comparable locations (Oinonen and Rouvinen, 2000). For example, the N-terminal nucleophile is nested in a characteristic αββα-fold, and highly conserved within the Ntn-Hydrolases superfamily (Brannigan et al., 1995; Oinonen and Rouvinen, 2000). This structure is providing the capacity for a nucleophilic attack and also enables the autocatalytic procession of the pro-enzyme (Hewitt et al., 2000).

Ntn-Hydrolases are synthesized as inactive precursor proteins that have to pass through a maturation process to be functional, as it was initially observed in PGA (Kasche et al., 1999). The precursor protein is composed of a signal peptide, an α-subunit and linker, and a β-subunit (Schumacher et al., 1986). During the maturation process, which is postulated to take place in the periplasm or during the translocation over the membrane, the N-terminal residue of the beta subunit is autoproteolyzed, separating the α- from the β-subunit and exposing the N-terminal nucleophilic residue to the solvent (Yoon et al., 2004). This is followed by a series of processing steps where the pro-region is sequentially removed from the C-Terminus of the α-subunit (Ignatova et al., 2005). However, the protein localization might not be essential for the proteins maturation and functionality, since the *pac* gene in *E. coli* without a signal sequence was found to be actively expressed in the cytosol (Xu et al., 2005). The linker or pro-peptide is masking the active site, including the nucleophilic residue (Hewitt et al., 2000). Comparison between the precursor and the mature protein, showed an extreme similarity of both structures, suggesting no major conformational changes occurred during the maturation process. Whereas it could be shown that the pro-peptide is crucial for the correct folding of the protein (Ignatova et al., 2005), it also brings together residues important for the autoproteolysis. In case of the AHL-acylase PvdQ, it could be shown that in the vicinity of the N-terminal nucleophile, a hydrophobic pocket is gated off from the solvent by a Phe(β24). This pocket is too small to fit the acyl chain of its ligand 3OC12-HSL. Upon binding, the pocket opens providing enough space for the binding (Bokhove et al., 2010).

The members of the protein family share the same amide bond hydrolysis mechanism. Initially the nucleophilic oxygen or sulfur of the nucleophile (Thr, Ser, and Cys) donates its proton to its own alpha amino group. Subsequently the nucleophilic attack on the carbonyl carbon of the substrate is launched, resulting in a tetrahedral intermediate, which in turn is stabilized by residues of the oxyanion hole (Peräkylä and Rouvinen, 1996). Afterward the α-amino group of the nucleophile donates its proton to the nitrogen of the scissile peptide bond, leading to a covalent bond with part of the substrate and the leaving amino product is released. In the second phase of the catalytic process, the acyl-enzyme complex will be cleaved in a deacylation step, returning the N-terminal nucleophile to its initial state. This is done by the hydroxyl group of a water molecule which attacks the carbonyl group of the acyl-enzyme complex (Duggleby et al., 1995).

### AHL-Acylases

**FIGURE 3:**
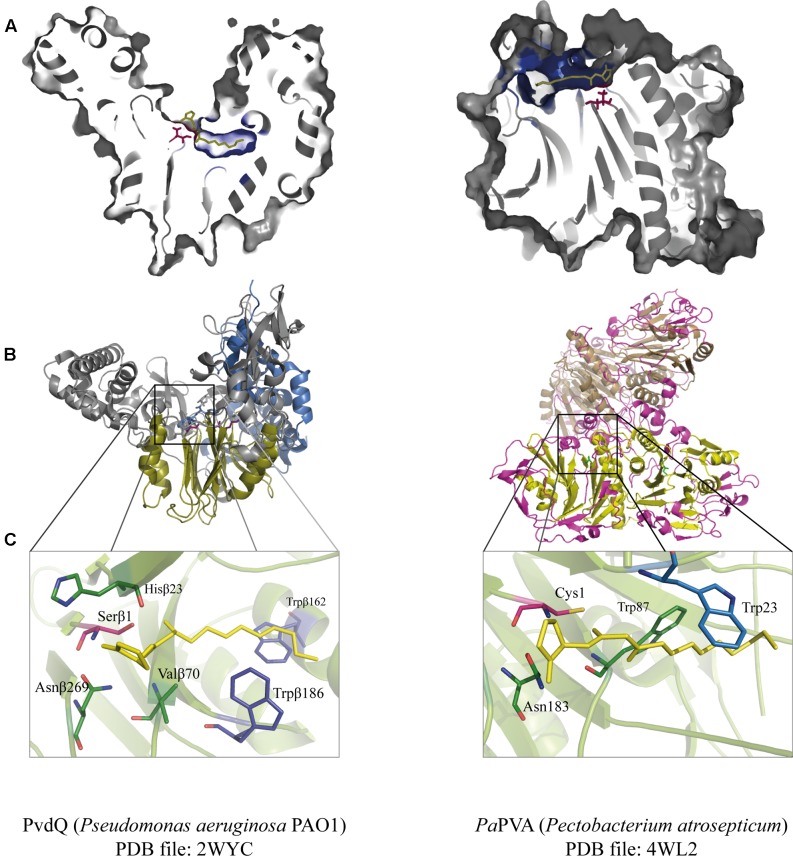
Juxtaposition of two representative members of the Ntn-hydrolase superfamily: PvdQ from *Pseudomonas aeruginosa* (left column) and *Pa*PVA from *Pectobacterium atrosepticum* (right column). **(A)** Cross-section of each enzyme showing the overview of the active sites with 3OC12-HSL as a substrate according to ligand docking data. The predicted binding pockets are colored in blue. **(B)** Structural comparison between the heterodimeric PvdQ (composed of subunit α and β) and the homotetrameric *Pa*PVA shows the presence of the characteristic αββα-fold, that are depicted in yellow ribbon. **(C)** Close-Up of the active centers of PvdQ (serine as the active nucleophile) and *Pa*PVA (cysteine as the active nucleophile). These enzymes share a striking resemblance in the active sites, showed here by the presence of the oxyanion hole that is crucial for the stabilization of the tetrahedral intermediate (PvdQ: Valβ70, Asnβ269, and Hisβ23; *Pa*PVA: Asn183 and Trp87). In addition, two tryptophan residues are thought to be important for the appropriate orientation of the substrate by providing a hydrophobic pocket for the acyl chain (PvdQ: Trpβ162 and Trpβ187; *Pa*PVA: Trp23 and Trp87). Ligand docking was performed in Discovery Studio 3.0 (Accelrys, United States) for PvdQ, and Glide’s extra precision for *Pa*PVA (Sunder et al., 2016). All structural figures were prepared in Pymol NRG studio.

The first enzymatic activity that involved deacylation of AHL was observed in *Variovorax paradoxus* VAI-C, in which this strain was found to be capable of assimilating AHL as a sole nitrogen and energy source (Leadbetter and Greenberg, 2000). This observation was soon followed by the isolation and characterization of the AiiD acylase from *Ralstonia* sp. that shared similarities with Ntn-hydrolases, such as aculeacin A acylase (AAC) and cephalosporin acylases (Lin et al., 2003). This finding stimulated the succeeding experiments of testing the ability of different enzymes from the Ntn-hydrolase family for hydrolysing AHLs. Gene PA2385 from *P. aeruginosa* was initially identified as β-lactam acylase based on sequence similarities with glutaryl acylase from *Pseudomonas* SY-77. The gene which is also referred as *pvdq*, displays all the characteristics of the Ntn-hydrolase superfamily such as the characteristic αββα-fold (**Figure 3B**) and it is synthesized as a precursor protein which undergoes an autocatalytic maturation step. PvdQ acylase is actively hydrolysing long-chain AHLs, but has no activity toward β-lactam compounds such as cephalosporin and penicillin (Sio et al., 2006). On the other hand, the glutaryl acylase SY-77 was not active with the AHLs, suggesting different substrate specificities for these enzymes.

The similar mode of action of AHL-acylase and penicillin acylases, leads to the idea of cross-reactivity between these enzymes (Krzeslak et al., 2007), and recent studies have confirmed this idea. *Kc*PGA from *Kluyvera citrophila* was found to be active on AHLs with 6–8 carbons with or without 3-oxo modification (Mukherji et al., 2014). Two PVAs from Gram-negative bacteria, namely *At*PVA (from *Agrobacterium tumefaciens*) and *Pa*PVA (from *Pectobacterium atrosepticum*) were found to be active on medium to long chain AHLs (Sunder et al., 2016). PVA and PGA differ in sequence, structural composition and N-terminal nucleophile residue. Similar to PvdQ, bacterial PGAs are generally heterodimeric with a serine as the Ntn residue, while bacterial PVAs are homotetrameric with cysteine as the Ntn residue. *In silico d*ocking showed a suitable fit of long-chain AHLs in the crystal structure of *At*PVA and *Pa*PVA (Sunder et al., 2016). On the other side, AhlM, an extracellular AHL-acylase from *Streptomyces* sp. was found to be able of cleaving penicillin G (Park et al., 2005). PA0305 from *P. aeruginosa* encodes HacB, an AHL-acylase that shows low activity toward penicillin V and penicillin G (Wahjudi et al., 2011). As mentioned in the previous section, despite of having low sequence similarity, the members of this superfamily share a comparable structure of the active site. Using PvdQ and PaPVA as AHL-acylase models that can degrade long-chain AHLs, the similarity of the orientation of the substrate to the N-terminal nucleophile and an acyl side chain binding pocket in both structures is obvious (**Figure 3**).

**FIGURE 4:**
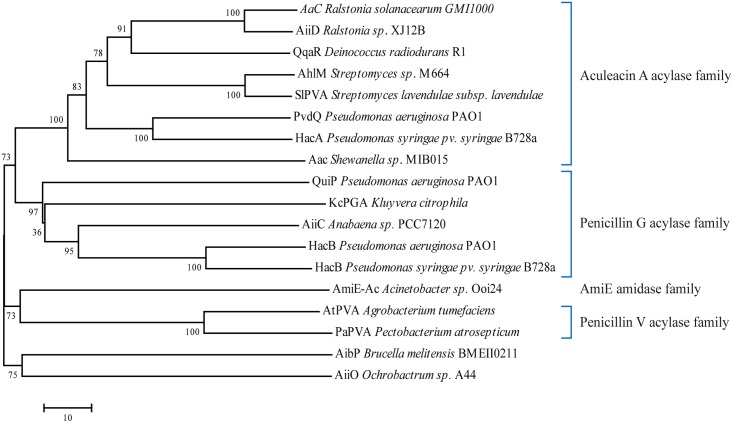
Phylogenetic tree from the known protein sequences of bacterial AHL-acylases. The phylogenetic tree was constructed using the neighbor-joining method with the ClustalW (MEGA program, ver. 7). Number represents Bootstraps values based on 1000 replications.

To date, many enzymes have been characterized and found to possess AHL-deacylating activity (**Table 1**). It is important to note that some enzymes in this list are produced by organisms lacking a AHL-dependent QS system (Gram-positive bacteria and eukarya). Phylogenetic analysis of the known bacterial AHL-acylases show four major groups, they are: AAC, PGA, PVA, and AmiE amidase family (Ochiai et al., 2014), with the exception of AiiO and AibP acylases that do not belong to any of these groups (**Figure 4**). We believe that this list is only a small portion of the abundant number of AHL-acylases that are waiting to be revealed. It is noteworthy to mention that kinetic data on AHLs are scarce due to the low solubility of most substrates. Nevertheless, recently quite some valid numbers have been reported and catalytic efficiencies in the order of 1–10 × 10^4^ per mole/min have been reported (e.g., HacB: 7.8 × 10^4^ M^-1^s^-1^, Wahjudi et al., 2011), which equals the best efficiencies reported on β-lactams (e.g., *Kc*PGA: 3.4 × 10^4^ M^-1^s^-1^, Mukherji et al., 2014).

**Table 1:** Widespread of AHL-acylases.

Source	AHL-acylase	Substrates^#^	Localization	Reference
**Prokarya**				
*Acinetobacter* sp. *Ooi24*	AmiE	3O10-HSL	n.i.	Ochiai et al., 2014
*Agrobacterium tumefaciens*	*At*PVA	(3O)C6-12 HSL	n.i.	Sunder et al., 2016
*Anabaena* sp. *PCC7120*	AiiC	3OC4-C14-HSL	Cell-associated, SP	Romero et al., 2008
*Brucella melitensis*	AibP	C12-HSL, 3OC12-HSL	Cell-associated	Terwagne et al., 2013
*Comamonas* sp. *strain D1*	n.i.	(3O)C4-C16-HSL	n.i.	Uroz et al., 2007
*Deinococcus radiodurans*	QqaR	(3O)C8-C14-HSL	n.i.	Koch et al., 2014a
*Kluyvera citrophila*	*Kc*PGA	(3O)C6-C8-HSL	n.i.	Mukherji et al., 2014
*Ochrobactrum* sp. *A44*	AiiO	(3O)C4-C14-HSL	Cytoplasmic	Czajkowski et al., 2011
*Pectobacterium atrosepticum*	*Pa*PVA	C10-HSL; C12-HSL	n.i.	Sunder et al., 2016
*Pseudomonas aeruginosa*	PvdQ	(3O)C10-C14-HSL	SP	Sio et al., 2006
	QuiP	(3O)C7-C14-HSL	SP	Huang et al., 2006; Wahjudi et al., 2011
	HacB	C6-C14-HSL	SP	Wahjudi et al., 2011
*Pseudomonas syringae strain B728a*	HacA	C8-HSL; C10-HSL; C12-HSL	Extracellular	Shepherd and Lindow, 2009
	HacB	(3O)C6-12-HSL	Cell-associated	Shepherd and Lindow, 2009
*Ralstonia mannitolilytica str. SDV*	n.i.	3OC10-HSL	n.i.	Yang et al., 2006
*Ralstonia solanacearum GMI1000*	Aac	C7-HSL; C8-HSL, 3OC8-HSL; C10-HSL	Cell-associated, SP	Chen et al., 2009
*Ralstonia* sp. *XJ12B*	AiiD	3OC8-HSL, 3OC10-HSL; 3OC12-HSL	SP	Lin et al., 2003
*Rhodococcus erythropolis* W2^∗^	n.i.	C4-HSL, C6-HSL, 3OC6-HSL, C7-HSL, C8-HSL, 3OC8-HSL, C10-HSL	n.i.	Uroz et al., 2003
*Shewanella* sp.	n.i.	C8-HSL, 3OC8-HSL, 3OHC8-HSL; C10-HSL; 3OC10-HSL; 3OHC10-HSL; C12-HSL; 3OC12-HSL; 3OHC12-HSL; C14-HSL	n.i.	Tait et al., 2009
*Shewanella* sp. *strain MIB015*	Aac	C8-HSL; C10-HSL; C12-HSL	SP	Morohoshi et al., 2008
*Streptomyces* sp. *strain M664*	AhlM	C8-HSL; C10-HSL; 3OC12-HSL	Extracellular	Park et al., 2005
*Streptomyces lavendulae* ATCC13664	SlPVA	n.d.	n.i.	Torres-Bacete et al., 2015
*Tenacibaculum maritimum strain NCIMB2154(T)*	n.i.	C10-HSL	Cell-associated	Romero et al., 2010
*Variovorax paradoxus VAI-C*	n.i.	C4-HSL, C6-HSL, 3-oxo-C6-HSL, C8-HSL, C10-HSL, C12-HSL, C14-HSL	n.i.	Leadbetter and Greenberg, 2000
**Eukarya**				
*Arabidopsis thaliana*	*At*FAAH	OC10, C12, OC12, OHC12, D-OC12, C12-HS, OC14	n.i.	Palmer et al., 2014
*Sus* sp. (porcine kidney)	pAcy1	C4-HSL; C8-HSL^∗∗^	n.i.	Xu et al., 2003


*n.i., not identified; SP, putative signal peptides. ^#^ see Supplementary Table S1.*

*^∗^Type of QQ enzymes production is strain-specific. *R. erythropolis* W2 produces acylase, lactonase and oxidase, while in *R. erythropolis* strains LS31 and PI33, only lactonase activity was observed (Park et al., 2006).*

*^∗∗^Last et al. (2016) indicated that pAcy1 shows no hydrolysing activity toward C6-HSL but toward C6-homoserine.*

*With the exception of *Ochrobactrum* sp. A44 AiiO, which has been classified as an αβ-hydrolase with a catalytic triad, all the other characterized bacterial enzymes belong to the Ntn-hydrolase family.*

## What Are the Potential Physiological Roles of AHL-Acylases?

After two decades since the earliest finding of AHL-acylase and the myriad of discoveries of novel acylases later on, the actual physiological roles of these enzymes are still obscure. With respect to their AHL-hydrolysing ability, these enzymes are speculated to be involved in the degradation of AHLs signaling molecules in nature. When a certain enzyme fulfills this proposed activity, it may lead to either of the following consequences: (i) AHL-degradation for fine tuning the endogenous QS system, (ii) AHL-degradation for modulating the QS system of other bacterial species, or even the microbiota where the acylases producers reside, (iii) AHL-degradation as a mechanism to use AHLs for nutrient sources, or to escape the toxicity of AHLs. Nonetheless, it is also possible that the natural substrates for certain acylases are not AHLs, and these enzymes are involved in biological systems unrelated to QS. In this section, we discuss the potential physiological functions of AHL-acylases based on the abovementioned points.

### Quorum Sensing Modulation

#### Intraspecies QS Modulation

*Pseudomonas aeruginosa* is a multihost pathogen that can be found in various environments and is mainly known as an opportunistic pathogen in immunocompromised patients and cystic fibrosis patients. The core QS system of this bacteria consists of the hierarchical LasRI and RhlRI systems, with 3OC12-HSL and C4-HSL as their cognate AHL, respectively (Venturi, 2006). Next to the QS system, four Ntn-hydrolase homologs are present in *Pseudomonas aeruginosa* PAO1*:* namely *pvdQ* (PA2385), *quiP* (PA1032), *hacB* (PA0305), and PA1893, in which the first three have been characterized as AHL-acylases (Huang et al., 2006; Sio et al., 2006; Wahjudi et al., 2011), while PA1893 is known to be part of QS-regulons (Schuster et al., 2003; Wagner et al., 2003). These enzymes efficiently hydrolyse long-chain AHLs, including the native 3OC12-HSL of *P. aeruginosa*. When grown in a rich medium, *P. aeruginosa* strains with constitutive expression of these acylases show a decreased level of 3OC12-HSL in the supernatant. The production of QS-regulated virulence factors elastase and pyocyanin was diminished in the strain overproducing HacB. In the strain overproducing PvdQ, reduction of elastase, pyocyanin and PQS levels was observed (Sio et al., 2006; Wahjudi et al., 2011). Vice versa, disruption of the acylase genes resulted in higher accumulation of AHL. A Δ*hacB* single mutant and a Δ*pvdQ*,Δ*hacB*,Δ*quiP* triple mutant accumulated 3OC12-HSL to a higher level compared to Δ*pvdQ*,Δ*quiP* double mutant that produced 3OC12-HSL in the same level as wild-type (Wahjudi et al., 2011). This observation indicates that in rich medium, HacB might be working as the main acylase in controlling long-chain AHL accumulation. The other explanation is that PvdQ and QuiP are produced only when a particular environmental cue(s) is present, as described below. The substrate specificity toward the AHL acyl chain length is determined by a hydrophobic pocket, which is interacting with the acyl chain. In a structure-aided design approach to modify the specificity of PvdQ this interaction was successfully demonstrated. By altering the structure of the hydrophobic pocket it was possible to effectively facilitate the binding and cleaving of the shorter C8-HSL (Koch et al., 2014b).

The *pvdQ* gene lies in the pyoverdine operon, which is responsible for biosynthesis of the main siderophore of *P. aeruginosa*. PvdQ works in one of the final maturation step of pyoverdine in the periplasmic space, in particular by removing myristoleic acid from the precursor PvdIq, generating ferribactin (Drake and Gulick, 2011). The crystal structure of PvdQ reveals a large hydrophobic binding pocket that serves its function to cleave a long-chain fatty acid (Bokhove et al., 2010). Pyoverdine is only produced in iron-limiting conditions, the environmental cue for pyoverdine biosynthesis gene to be expressed. However, it is important to note that a *pvdQ* deletion strain grown in iron-limiting media did not only show a defect in pyoverdine production, but also accumulated higher concentrations of 3OC12-HSL compared to wild-type (Nadal Jimenez et al., 2010), supporting the indication of a dual function for PvdQ in *P. aeruginosa*. Among the described acylases, QuiP is crucial in assimilation of long-chain AHLs as carbon source (Huang et al., 2006). A Δ*quiP* deletion mutant showed a growth defect when utilizing AHLs as carbon source. Microarray analysis from cultivating the bacteria in a medium with C10-HSL as a sole carbon source, showed an up-regulation of *quiP* and PA1893, but did not affect *pvdQ* and *hacB* expressions. Taken together, this observation hints to the possibility that QuiP functions in AHL-scavenging (Krzeslak et al., 2007).

*Brucella melitensis* is a Gram-negative infectious agent of Brucellosis, a zoonotic disease that is transmitted via unpasteurized dairy products or direct contact with the infected animals (Moreno, 2014). The bacterium possesses LuxR homologs, namely VjbR and BabR that are known as global regulators for virulence factors production and stress response (Uzureau et al., 2007). A low concentration of C12-HSL (and possibly 3O-C12-HSL) was detected *in vitro*, while the synthase(s) currently have not been characterized yet (Taminiau et al., 2002). The QS system works in a non-classical manner, in which the produced C12-HSL negatively influences the production of virulence factor, specifically the expression of flagellar genes.

Using an elegant AHL-sensitive reporter system (Hentzer et al., 2002) that allows observation of C12-HSL accumulation at a single bacterium level, the study revealed another hint of this QS regulatory mechanism (Terwagne et al., 2013). The intracellular accumulation of C12-HSL *in vitro* was reduced at the end of logarithmic phase when the population density was the highest. It means that the accumulation of C12-HSL in this bacterium does not reflect the increase of population density, unlike the majority of the known QS system. This phenomenon is suspected to be mediated by a QQ enzyme named AibP acylase. This enzyme has a conserved region at the predicted α and β subunit as it is a member of the Ntn-hydrolase superfamily. In the wild-type strain, the peak of *aibP* expression coincides with the decrease of intracellular C12-HSL level. In agreement with this observation, disruption of the *aibP* gene resulted in the tenfold higher C12-HSL accumulation both *in vitro* and during macrophage infection. In the Δ*aibP* mutant, a decrease of flagellar genes expression was observed to the same level as C12-HSL addition to the wild-type strain. These observations show that the modulation of C12-HSL concentration by AibP acylase favors virulence factors production.

#### Modulating a Multispecies Community

Highly complex microbial assemblies called microbial granules or granular sludge are emerging as effective methods for wastewater treatment (Liu and Tay, 2004). Granular sludge consists of aggregates of numerous microorganisms formed by self-immobilization that exhibit complicated interactions. Granular sludge is cultivated in aerobic conditions leading to strong and robust particles able to perform biodegradation of toxic wastes such as phenols (Jiang et al., 2002; Tay et al., 2005; Liu et al., 2009). Aerobic granules are produced in sequential batch reactors (SBR) seeded by activated sludge (Liu and Tay, 2004; Adav et al., 2008). Tan et al. (2014, 2015) revealed the involvement of AHL-dependent QS that is modulated by QQ during the granulation cycle. In the first study, short to medium-chain AHLs predominated by 3OC8-HSL, were found to have accumulated during granules formation. Conversely, the dispersion of mature granules coincided with the decrease of the AHL accumulation below detection level (Tan et al., 2014). This observation is in agreement with previous finding that AHLs and autoinducer-2 (AI-2, a signaling molecule in Gram-positive bacteria) are important in maintaining the structural integrity of aerobic granules (Jiang and Liu, 2013). Supplementation of exogenous AHLs induced aggregation of floccular biomass, suggesting the importance role of AHL level in the formation of granules.

16S rDNA analysis from this microbial community, showed a presence of both AHL-producing organisms (from α, β, and γ Proteobacteria) and QQ organisms (from phyla Proteobacteria, Actinobacteria, Bacteroidetes, and Firmicutes), wherein some are both AHL-producers and AHL-degraders (Tan et al., 2015). Based on isolation studies, floccular sludge communities were predominantly QQ organisms (60%), in which more than 35 organisms were in the top 50 most abundant community members. Among these QQ organisms, the previously known genera that produce AHL-acylase or AHL-lactonase were isolated, such as *Variovorax*, *Bacillus, Ochrobactrum*, and *Rhodococcus*. Almost all QS-proficient organisms in floccular sludge were capable of producing long-chain AHLs and these AHLs were also the preferred substrates for all QQ organisms. Hence, only short to medium-chain AHLs were detected at low concentration at this phase. It is important to note that AHLs degradation in this complex community is attributed to the enzymatic degradation activity rather than spontaneous hydrolysis. Interestingly, the microbial consortia during granules formation were dominated by AHL-producing organisms, explaining the increase of AHL accumulation at this stage (Tan et al., 2015). This observation demonstrates the important role of AHLs signal fine-tuning to direct community behavior. This modulation of AHLs might also occur in natural environments where complex and dynamic multispecies communities exist.

#### Modulating Interkingdom Interactions

Biofouling is an accumulation of microorganisms, algae, plants, or animals on wetted artificial surfaces, causing serious problems in marine industry and underwater structures. The most common macroalga associated with biofouling is from the genus *Ulva*. Asexual reproduction of *Ulva* involves a release of motile zoospores from mature algae, followed by a quest for new suitable surfaces to settle. Once a zoospore locates an appropriate site, it produces adhesive-containing vesicles to support its permanent attachment, and subsequently grows into the mature organism (Callow and Callow, 2000). The settlement itself depends on multiple environmental cues, including the presence of bacterial biofilms (Taylor et al., 2000). AHLs released by the biofilms attract the zoospores and induce calcium influx that relates to the modulation of flagellar movement resulting in a decrease in swimming rate (Joint et al., 2007). The zoospores can detect a wide range of AHLs, preferably with acyl chains longer than six carbons (Tait et al., 2005).

Further investigation of bacterial communities and AHL modulation *in situ* was performed by Tait et al. (2009). Samples of intertidal rock-pools colonized by *Ulva* were collected from Wembury beach, United Kingdom. LC/MS-MS analysis from the dichloromethane extract of the pebbles showed a presence of AHLs, including C8-HSL and C10-HSL. These AHLs are believed to be produced by some members of the bacterial community colonizing the rocks, confirmed by metagenomic analysis and laboratory cultures. Among these species were the previously unknown *Sulfitobacter* (α-Proteobacteria family), as well as *Glaciecola* and *Marinobacteria* (Alteromonadaceae family). An identified isolate of *Shewanella* sp. is both an AHL-producing (3OC4-HSL, 3OC10-HSL, and 3OC12-HSL) and AHL-degrading strain (acylase activity to medium until long chain AHLs). *In vitro* experiments of unispecies biofilms with variation in biofilm density, showed a preference of the zoospores to settle on the biofilm with the highest AHL concentration. AHL-acylases enzymes in *Shewanella* sp. modulate its AHL concentration, hence affecting the zoospores settlement. Interestingly, this effect was also shown in the multispecies biofilm of *Vibrio* and *Sulfitobacter*, in which the co-culturing with *Shewanella* sp. reduces the zoospores settlement. These observations suggest that the presence of AHL-degrading species plays a role in AHL turnover within a microbial community, which in turn influences interkingdom interaction, as seen with the *Ulva* zoospores settlement.

The abovementioned examples of *Ulva* with marine bacteria, and plants with their rhizhobia symbionts confirm that plants are capable of recognizing AHLs. However, there is a lack of knowledge of how plants perceive these AHLs. Using young seedlings of plant model *Arabidopsis thaliana* and *Medicago truncatula*, Palmer et al. (2014) show a biphasic effect of AHLs in primary root growth of the plants in a concentration-dependent manner. Low concentrations of AHLs induce primary root growth via transpiration that induces nutrient uptake, and in contrast, higher concentrations induce an adverse effect of growth inhibition, due to ethylene production. The response was more pronounced for the AHLs with acyl chain of 12 carbons or longer. Interestingly, exposure to L-homoserine (HS), but not to a long-chain acyl tail, elicited a comparable response as an exposure to C12 to C14-HSLs. This result indicated the occurrence of AHLs deacetylation to release L-homoserine lactone (HSL), which is further hydrolyzed into HS, a signal that is recognized by the plants. An enzyme called fatty acid amide hydrolase (FAAH) produced by diverse plants and animals species was suggested to be responsible for this function (Ortíz-Castro et al., 2008; Palmer et al., 2014). FAAH is a serine hydrolase that belongs to the amidase signature family. This enzyme is part of a system that regulates the endogenous concentration of *N*-acylethanolamines, a fatty acid derivatives group important in an array of physiological and behavioral functions (Chapman et al., 2006). Purified FAAH from *A. thaliana* (*At*FAAH) cleaved the same type of AHLs that are modulating the growth *in planta*. The hypothesis was further confirmed by a Δ*At*FAAH deletion mutant that was shown to be less sensitive to the exposure of AHLs. It is clear that the presence of FAAH is important in perceiving AHLs in the plant models.

### AHL Turnover Mechanism

In order to serve as a “signal,” the signaling molecules not only have to be produced and accumulated at a certain moment, but they also have to be abolished when the signaling is no longer required. Hence, AHLs turnover mechanisms should be existing as a way to avoid the build-up of these molecules, and allow a proper utilization of the signaling molecules in reflecting the population density or as an environmental cue. AHLs are known to undergo a non-enzymic lactonolysis at high pH and at increased temperature, in which the AHLs with longer acyl chains are more stable (Yates et al., 2002). Nevertheless, enzymatic degradation of AHL in nature is also believed to occur and has been speculated as one of the natural roles of QQ enzymes.

The rhizosphere is home for a rich diversity of microorganisms, including those that are capable of degrading AHLs. Bulk soil samples are able to biologically mineralize AHL with high efficiency, in which the degradation products most probably are utilized to form biomass by the microorganisms (Wang and Leadbetter, 2005). In fact, the first bacterium with AHL-acylase activity (*V. paradoxus* VAI-C) was isolated from turf soil (Leadbetter and Greenberg, 2000). This bacterium is able to utilize AHLs, HS, and HSL as sole nitrogen sources, but only the first two substrates can be utilized as a carbon source. The AHLs metabolism pathway is still unknown, but the deacylation of AHL is believed to be the first step to release the acyl chain, which can be processed further. Another bacterium that was isolated from the same enrichment culture, *Arthrobacter* strain VAI-A, showed an aberrant growth in defined media with 3OC6-HSL, but metabolized a substrate with an opened lactone ring 3OC6-HS (as carbon and nitrogen source) and HSL (as nitrogen source) (Flagan et al., 2003). Interestingly, a co-culture of *V. paradoxus* with *Arthrobacter* VAI-A in a defined medium with 3OC6-HSL as a sole carbon and nitrogen source significantly increased the growth rates compared to monocultures in the same medium composition. The nutrient symbiosis between these bacteria was not fully understood, but it is speculated that the remaining carbon from the utilized HSL is used as an energy source for *Arthrobacter* VAI-A.

An enrichment culture of a different soil sample revealed other strains of *Arthrobacter* species (strain HSL-1, HSL2, and HSL-3), and *Burkholderia* strain HSL-4 that can utilize HSL as a sole carbon and nitrogen source (Yang et al., 2006). As predicted, the AHL dependent growth rates were stimulated during the co-culture between these HSL-degrading strains and AHL-acylase producer *Ralstonia mannitolilytica* strain SDV. These observations indicate the presence of AHL-degrading and AHL-mineralizing consortia in the soil, explaining why accumulation of AHLs is scarcely found in the soil.

### Escaping AHLs-Mediated Toxicity

The AHLs not only function as signaling molecules, but also show a potent antibacterial activity (Kaufmann et al., 2005). AHLs with an oxo-group substitution at the third carbon (3O-AHLs) in aqueous environment can undergo spontaneous hydrolysis into acylhomoserines and tetramic acids (Kaufmann et al., 2005). The latter compounds display strong antibacterial activity toward Gram-positive bacteria, but are tolerated by Gram-negative bacteria. Interestingly some Gram-positive bacteria have the machinery to degrade AHLs. Therefore in Gram-positive bacteria, which are not using AHLs as signaling molecules, AHL-degrading enzymes can give a competitive advantage in bacterial warfare. Consequently some strains of *Bacillus cereus* and *B. thuringiensis* producing AiiA lactonase show resistance toward 3O-AHLs. The AHL-degrading activity of these enzymes presumably protects the bacteria from 3OC12-HSL and prevent the conversion into tetramic acid derivative (Kaufmann et al., 2005). In a reciprocal study, a *B. thuringiensis* mutant defective in AiiA production displayed a decreased survival rate in a mixed culture with the plant pathogen *Erwinia carotovora* on the pepper root (Park et al., 2008). *E. carotovora* is also known to utilize AHL-dependent QS to regulate the exoenzyme and toxin production (Bainton et al., 1992). With the same rationale, it can be imagined that the Gram-positive soil bacterium *Streptomyces* spec. utilizes its extracellular AHL-acylases (AhlM) to confer the same advantages to the producing bacteria (Park et al., 2005).

## Concluding Remarks

The earliest discovery of AHL-degrading enzymes was immediately followed by postulations about their role in AHL turnover and QS modulation in nature. Despite of the inquisitiveness, research to explore the fundamental basis of why QQ enzymes exist was outcompeted by the enthusiasm of potential therapeutic and industrial applications of these enzymes. Nevertheless, toils in this area have been conducted to unveil some *in vivo* physiological functions of the QQ enzymes. AHL degradation is indispensable to prevent a signal build-up and to allow a real-time monitoring of *de novo* AHLs synthesis to reflect the population density. In the context of multispecies interactions, it is becoming apparent that signal fine tuning and quenching is as important as signal transmittance itself. A notable observation of a highly complex community showed that both QS and QQ play important roles in directing the community behavior, or indirectly modulating the behavior of other organism whose fate is depending on AHLs cues. Within a highly complex community, AHL degradation is counted as a cumulative function of populations rather than individuals, hence the performances of multiple AHL-hydrolysing enzymes are often entangled. In that respect, it should be noted that the action of lactonases can be reverted at low pH, whereas the degradation by AHL-acylases is irreversible.

*N*-Acylhomoserine lactones are more than merely communication signals; these molecules are multifunctional and can exert detrimental effects to other organisms. It is unsurprising that AHL-degrading enzymes are regarded as a part of the arsenal during bacterial warfare, given the bestowed benefit to the producing organisms. This advantage is not limited to bacteria only as mammalian paraoxonases (AHL-lactonases) can inactivate AHLs. These enzymes are considered to be defense mechanisms against bacterial pathogenesis (Ozer et al., 2005; Stoltz et al., 2007; Devarajan et al., 2013). However, considering the prevalence and the efficiency of the AHL-degrading enzymes, one might ask how the AHL-dependent QS systems are still successfully operated, in spite of the vulnerability of extracellular AHLs. Docking simulations of both PvdQ and *Pa*PVA demonstrate that AHLs fit perfectly in the binding pocket of the respective enzymes raising questions not about the design of the enzymes but the design of the signaling molecules itself. It is important to emphasize that AHLs do not represent the only autoinducers playing a signaling role in QS of Gram-negative bacteria. For instance, *P. aeruginosa* uses in addition to 3OC12-HSL and C4-HSL, a 2-heptyl-3-hydroxy-4-quinolone (PQS).

Enzymes are classified based on enzymatic activities they possess, or are predicted to be a member of a certain class based on a homology analysis or the presence of particular motifs. The classification system is robust and practical to easily point out certain activities an enzyme could catalyze. However, in many cases, enzymatic nomenclature could be a source of confusion when determining the native physiological role of the enzymes. This confusion applies to the names penicillin and cephalosporin acylase. The products of these enzymes, 6-APA and 7-ACA are more toxic to the enzyme producer than the substrates penicillin and cephalosporin C. Therefore, it is difficult to imagine a physiological function for these enzymes in potentiating β-lactams. A natural role in degrading AHL is better conceivable. On the other hand, considering the variability of individual enzyme characteristics and the multitude of producers, it is too pretentious to assign AHLs as the native substrate for *all* here-mentioned AHL-acylases. Some of the well-studied enzymes showed a broad range of substrate specificities, and it is challenging to point out which one is the natural substrate. The AHL-degrading activity could be complementary to the main function of the enzyme. For example, during iron-limiting conditions, PvdQ acylase in *P. aeruginosa* was observed to play a dual role: pyoverdine biosynthesis and regulating AHL concentration. This double function is also evident in BlcC lactonase (formerly AttM) produced by the plant tumor-inducing pathogen *Agrobacterium tumefaciens*. The BlcC lactonase controls the accumulation of endogenous 3OC8-HSL at the onset of tumor progression, and is also involved in the production of gamma-hydroxybutyrate (GHB) from gamma-butyrolactone (GLB) (Zhang et al., 2002; Khan and Farrand, 2009).

Hitherto, the gained knowledge of QQ enzymes (AHL-acylases in this review) is only the tip of the iceberg. There are still countless unidentified enzymes and mechanisms awaiting to be discovered.

## Author Contributions

WQ initiated the research on penicillin and cephalosporin acylases. He directed research toward the AHL degrading activity of this class of enzymes. PU has performed several project on the quorum quenching activity of AHL acylases and she has read and interpreted many of the cited references. JV has collected literature data and written part of the manuscript.

## Conflict of Interest Statement

The authors declare that the research was conducted in the absence of any commercial or financial relationships that could be construed as a potential conflict of interest.
