# Supplementary material for: Deciphering Physiological Functions of AHL Quorum Quenching Acylases
Source: Front Microbiol. 2017 Jun 19;8:1123. doi: 10.3389/fmicb.2017.01123 (PMC5474475; doi:10.3389/fmicb.2017.01123)
Supplement: Supplementary file 1 [file Table_1.DOCX]

Supplementary Material

Deciphering physiological functions of quorum quenching acylases

Putri Dwi Utari, Jan Vogel, Wim J. Quax*

*** Correspondence:** Wim J. Quax: w.j.quax@rug.nl

**Table S1**. List of abbreviated molecules

| **Abbreviation** | **AHLs** | **IUPAC** |
| --- | --- | --- |
| C4-HSL | *N*-butanoyl-L-homoserine lactone | *N*-(Tetrahydro-2-oxo-3-furanyl)-butanamide |
| C6-HSL | *N*-hexanoyl-L-homoserine lactone | *N*-(Tetrahydro-2-oxo-3-furanyl)-hexanamide |
| C7-HSL | *N*-heptanoyl-L-homoserine lactone | *N*-(Tetrahydro-2-oxo-3-furanyl)-heptanamide |
| C8-HSL | *N*-octanoyl-L-homoserine lactone | *N*-(Tetrahydro-2-oxo-3-furanyl)-octanamide |
| C10-HSL | *N*-decanoyl-L-homoserine lactone | *N*-(Tetrahydro-2-oxo-3-furanyl)-decanamide |
| C12-HSL | *N*-dodecanoyl-L-homoserine lactone | *N*-(Tetrahydro-2-oxo-3-furanyl)-dodecanamide |
| C14-HSL | *N*-tetradecanoyl-L-homoserine lactone | *N*-(Tetrahydro-2-oxo-3-furanyl)-tetradecanamide |
| C14:1HSL | *N-*tetradecenoyl-L-homoserine lactone | *N-(Tetrahydro-2-oxo-3-furanyl)-9-cis-tetradecenamide* |
| C16-HSL | *N*-hexadecanoyl-L-homoserine lactone | *N*-(Tetrahydro-2-oxo-3-furanyl)-octanamide |
| C16:1HSL | *N*-hexadecenoyl-L-homoserine lactone | *N*-(Tetrahydro-2-oxo-3-furanyl)-9-cis-hexadecenamide |
| C18-HSL | *N*-octadecanoyl-L-homoserine lactone | *N-(Tetrahydro-2-oxo-3-furanyl)-octadecanamide* |
| 3OC6-HSL | *N*-(3-oxohexanoyl)-L-homoserine lactone | 3-Oxo-*N*-(tetrahydro-2-oxo-3-furanyl)-hexanamide |
| 3OC8-HSL | *N*-(3-oxooctanoyl)-L-homoserine lactone | 3-Oxo-*N*-(tetrahydro-2-oxo-3-furanyl)-octanamide |
| 3OC10-HSL | *N*-(3-oxodecanoyl)-L-homoserine lactone | 3-Oxo-*N*-(tetrahydro-2-oxo-3-furanyl)-decanamide |
| 3OC12-HSL | *N*-(3-oxododecanoyl)-L-homoserine lactone | 3-Oxo-*N*-(tetrahydro-2-oxo-3-furanyl)-dodecanamide |
| 3OC14-HSL | *N*-(3-oxotetradecanoyl)-L-homoserine lactone | 3-Oxo-*N*-(tetrahydro-2-oxo-3-furanyl)-tetradecanamide |
| 3OC16:1-HSL | *N*-(3-oxohexaadecenoyl)-L-homoserine lactone | 3-Oxo-*N*-(tetrahydro-2-oxo-3-furanyl)-9-cis-hexadecenamide |
| HSL | L-homoserine lactone |  |
| HS | L-homoserine |  |
